# A mucin protein predominantly expressed in the female-specific symbiotic organ of the stinkbug *Plautia stali*

**DOI:** 10.1038/s41598-022-11895-1

**Published:** 2022-05-11

**Authors:** Minoru Moriyama, Toshinari Hayashi, Takema Fukatsu

**Affiliations:** 1 Bioproduction Research Institute, National Institute of Advanced Industrial Science and Technology (AIST), Tsukuba, 305-8566 Japan; 2 Department of Biological Sciences, Graduate School of Science, University of Tokyo, Tokyo, 113-0033 Japan; 3 Graduate School of Life and Environmental Sciences, University of Tsukuba, Tsukuba, 305-8572 Japan

**Keywords:** Evolution, Molecular biology

## Abstract

Diverse insects are obligatorily associated with microbial symbionts, wherein the host often develops special symbiotic organs and vertically transmits the symbiont to the next generation. What molecular factors underpin the host-symbiont relationship is of great interest but poorly understood. Here we report a novel protein preferentially produced in a female-specific symbiotic organ of the stinkbug *Plautia stali*, whose posterior midgut develops numerous crypts to host a *Pantoea*-allied bacterial mutualist. In adult females, several posteriormost crypts are conspicuously enlarged, presumably specialized for vertical symbiont transmission. We detected conspicuous protein bands specific to the female’s swollen crypts by gel electrophoresis, and identified them as representing a novel mucin-like glycoprotein. Histological inspections confirmed that the mucin protein is localized to the female’s swollen crypts, coexisting with a substantial population of the symbiotic bacteria, and excreted from the swollen crypts to the midgut main tract together with the symbiotic bacteria. Using RNA interference, we successfully suppressed production of the mucin protein in adult females of *P. stali*. However, although the mucin protein was depleted, the symbiont population persisted in the swollen crypts, and vertical symbiont transmission to the next generation occurred. Possible biological roles and evolutionary trajectory of the symbiosis-related mucin protein are discussed.

## Introduction

Stable vertical transmission of mutualistic microorganisms underlies the evolution of intimate and sophisticated symbiotic systems among diverse insects, especially those relying on nutritionally imbalanced food resources, such as plant sap, vertebrate blood, and indigestible plant materials^1–7^. These intimate symbiotic relationships are often accompanied by the development of specialized symbiont-harboring organs^4,8^, symbiont genome reduction^9,10^, and insect-symbiont co-cladogenesis^5,11^.

Many stinkbugs of the superfamily Pentatomoidea have established mutualistic associations with specific symbiotic bacteria of the Enterobacteriaceae that are harbored in a specialized posterior region of the midgut^12–23^. The epithelium of the symbiotic midgut is structurally differentiated for the symbiotic association, bearing rows of numerous sac-like crypts for harboring the symbiotic bacteria. A number of studies have reported that experimental elimination of the gut symbionts causes deleterious effects on host development and reproduction, indicating mutualistic nature of the association^20,24–28^. Their primary contribution to host fitness is deduced as supplementation of nutrients such as essential amino acids and vitamins, which were inferred either from the gene repertoire of the symbiont genomes^21,29–33^ or from in vitro culture experiments of the midgut symbiotic organs^34^.

While endocellular symbionts are transovarially inherited to the next host generation within the maternal body, extracellular gut symbionts are generally passed to the host offspring postnatally, for which the symbionts must be excreted and survive outside the host body at least transiently^5,35^. For enabling survival under the harsh conditions outside the host, adult females of diverse stinkbugs embed the symbiont cells in a maternal secretion and deposit the symbiont-containing secretion alongside the eggs. The composition and configuration of the maternal secretion are diverse among stinkbug groups: symbiont-containing “capsules” in the Plataspidae^15,24,34,36,37^, symbiont-containing jelly-like substance in the Urostylididae^21^, symbiont-containing white mucus in the Parastrachiidae^38^, and, most prevalently, symbiont-containing secretion smeared on eggshell in the Pentatomidae^18,30,39,40^, the Cydnidae^26^, the Scutelleridae^20,41,42^, and the Acanthosomatidae^12^. In either format, the newborn nymphs orally acquire the secretion to establish gut colonization of the symbiotic bacteria^15,21,26,37,38,43^.

Besides the main symbiotic organ at the posterior midgut, some stinkbug groups were reported to develop additional distinct symbiont-containing organs or tissues such as yellow organs in the Urostylididae^21^, lubricating organs in the Acanthosomatidae^12^, capsule-producing organs in the Plataspidae^24,34,36^, and swollen crypts in the Pentatomidae and the Scutelleridae^41,44–47^, which are predicted to be involved in production of the symbiont-containing secretion for vertical symbiont transmission. While anatomical descriptions of such organs have been accumulated, their biochemical and molecular aspects have been very poorly investigated. The only molecular study was reported recently: in plataspid stinkbugs, a novel secretion protein with an odorant binding protein motif, which is produced by the female-specific capsule-producing organs, was identified as the primary constituent of the symbiont-containing capsules that enables symbiont survival outside the host and ensures vertical symbiont transmission^34^.

**Figure 1:**
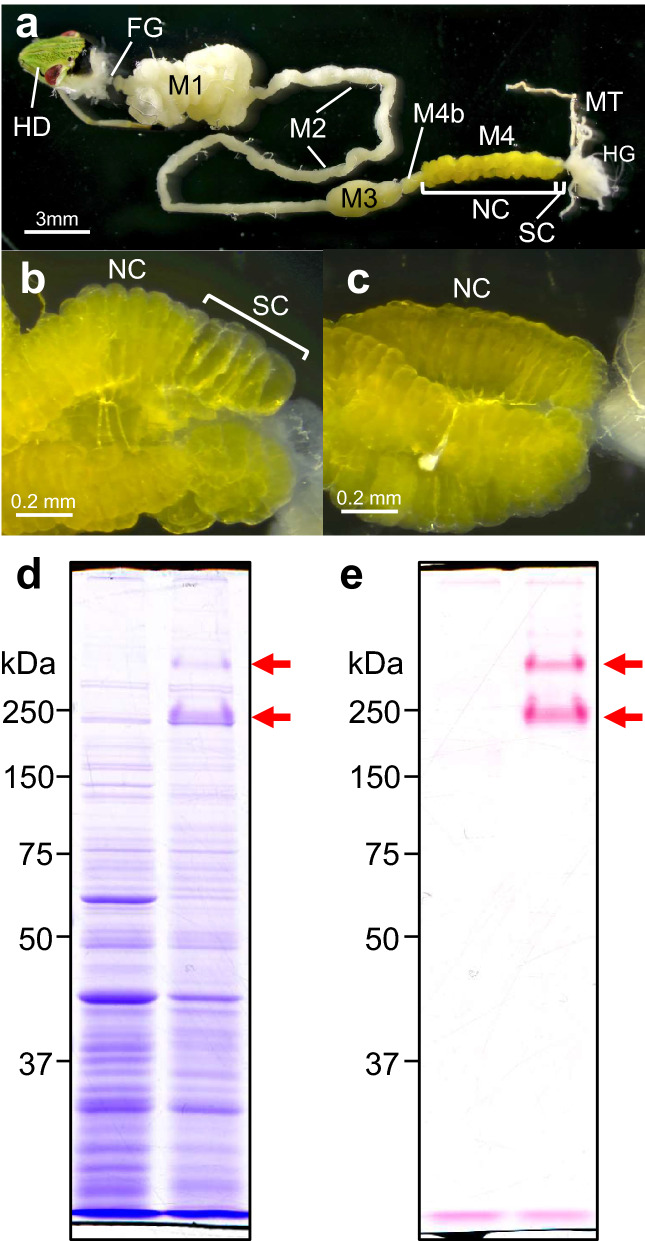
Detection of proteins specifically expressed in the female-specific swollen crypts of the symbiotic midgut. (**a**) An alimentary tract dissected from an adult female. The symbiotic midgut M4 region consists of a long stretch with normal crypts and a posteriormost short region with swollen crypts. (**b**) The posterior end of the midgut M4 region of an adult female with swollen crypts. (**c**) The same region of an adult male without swollen crypts. HD, head; FG, foregut; M1, M2 and M3, midgut M1, M2 and M3 regions; M4b, bulb-like midgut region anterior to M4; M4, symbiotic midgut M4 region with crypts; NC, normal crypt; SC, swollen crypt; HG, hindgut; MT, Malpighian tubule. (**d**,**e**) Proteins of normal crypts and swollen crypts were separated on SDS-PAGE gels and visualized by CBB staining for whole proteins (**d**) and PAS staining for glycosylated proteins (**e**). Arrows show the prominent protein bands specifically detected from the swollen crypts.

In this study, we investigated molecular aspects of the female-specific symbiont-containing organs in a pentatomid stinkbug *Plautia stali*. The brown-winged green stinkbug *P. stali* is obligatorily associated with *Pantoea*-allied gut symbiotic bacteria that are vertically transmitted via egg-surface smearing^18,48^. The midgut of *P. stali* consists of structurally distinct regions, M1, M2, M3, M4b and M4 from oral to aboral side, of which the posterior M4 region develops numerous crypts arranged in four rows and harbors the specific symbiotic bacteria inside (Fig. 1a)^49^. In adult females of pentatomid stinkbugs including *P. stali*, several crypts at the posterior terminal region exhibit distinct morphological traits, being enlarged and translucent in comparison with the anterior normal crypts (Fig. 1b,c)^46^. We compared the protein constituents of the female-specific swollen crypts with those of the normal crypts, identified a peculiar mucin-like glycoprotein predominant in the swollen crypts, and investigated its protein feature, expression profile, histological localization, and possible involvement in vertical symbiont transmission.

## Results

### Identification of swollen crypt-specific proteins

In an attempt to identify proteins that are specifically expressed in the females-specific swollen crypts, we first compared the protein profiles of the normal crypts and the swollen crypts dissected from adult females of *P. stali* by SDS-PAGE. Coomassie Brilliant Blue (CBB) staining of the SDS-PAGE gels revealed that the banding patterns were generally similar between the normal crypts and the swollen crypts, but two prominent protein bands were specifically detected in the swollen crypts, which were located at low mobility positions of apparent molecular mass around 200 kDa and 400 kDa (Fig. 1d). The edges of these bands were strongly stained and smiling upward. We found that these bands were preferentially detected by Periodic acid Schiff (PAS) staining (Fig. 1e), suggesting that they are sugar-conjugated proteins.

**Figure 2:**
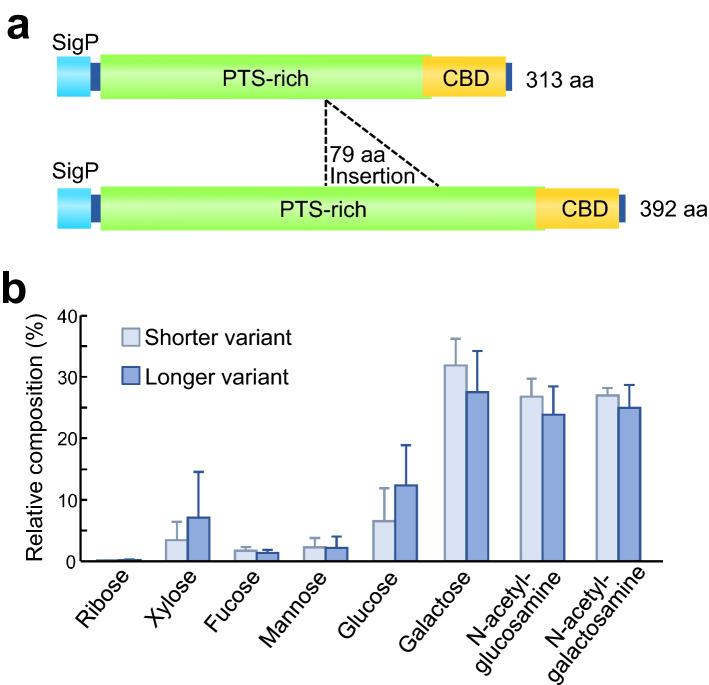
Swollen crypt-specific mucin-like glycoprotein designated as SC mucin. (**a**) Schematic structure of shorter variant and longer variant of the SC mucin proteins. The bars represent signal peptide domains (SigP), proline, threonine and serine-rich domains (PTS-rich), and chitin-binding domains (CBD). (**b)** Composition of monosaccharides liberated by acid hydrolysis of shorter variant and longer variant of the SC mucin proteins retrieved from SDS-PAGE gels (*N* = 3).

We performed LC–MS/MS-based identification of the PAS-positive proteins. Prior to the mass examination, we conducted RNA-sequencing and de novo gene assembly to establish a reference catalogue of proteins representing the symbiotic midgut. We analyzed each of two specific proteins that exhibited different mobility on SDS-PAGE gels (Fig. 1d) and found that, unexpectedly, both proteins were mapped to C-terminal region of the same protein record (TRINITY_DN2761_c1_g1_i1.p1) (Table 1). We determined the full-length mRNA sequences by PCR-amplification using several primer sets (Supplementary Fig. S1), and identified two sequences that shared the common 5′- and 3′-regions but differed in size. The shorter variant was 1138 bp long, while the longer variant contained a 237 bp additional sequence in the middle region. Note that several single nucleotide substitutions were found in the shared regions. These genes were deduced to produce 32.7 kDa and 39.9 kDa polypeptides that have a secretory signal motif at the N-terminus and a chitin-binding domain at the C-terminus (Fig. 2a). No similar proteins were found in the protein database by Blastp similarity search against non-redundant protein sequences of the National Center for Biotechnology Information (e-value cutoff = 1e^−10^). Notably, the major part of these proteins, including the inserted region of the longer variant, was occupied by a large number of potential O-glycosylated residues consisting of proline, threonine and serine (= PTS) (Fig. 2a, Supplementary Fig. S2). These three amino acid residues accounted for 68.5% (161/235) and 73.9% (232/314) of the PTS-rich domains for the shorter and longer variants, respectively. Such PTS-rich domains are known to be typical of mucin family glycoproteins^50^. When monosaccharide compositions of these proteins were analyzed by LC–MS after acid hydrolysis, canonical sugar components of mucin-type O-glycosylation, including N-acetylgalactosamine, N-acetylglucosamine and galactose^51,52^, were abundantly detected from both the variants (Fig. 2b). These features indicate that these proteins are heavily glycosylated mucin-like proteins. Hereafter, we designate this swollen crypt-specific mucin-like protein as SC mucin. On the ground that each insect contained either one or both of the short- and long-type SC mucins (Supplementary Fig. S3), it seemed likely that the two length forms represent allelic variants that arose from the same gene locus.

**Table 1:** Results of protein identification using LC–MS/MS.

Sample	Protein hit					Peptide hit			
	Protein accession	Predicted mass	Mascot score	No. peptide hit	Sequence coverage (%)	Peak ID	Start pos.	End pos.	Sequence
Longer	TRINITY_DN2671_c0_g1_i1	34,274	88	3	6.79	42	295	301	K.HGFWVHR.S
						47	308	315	K.CFDTVHLK.C
						30	318	324	K.FWNAICG.-
Shorter	TRINITY_DN2671_c0_g1_i1	34,274	62	2	4.94	63	308	315	K.CFDTVHLK.C
						55	318	324	K.FWNAICG.-

### Expression and localization of SC mucin

**Figure 3:**
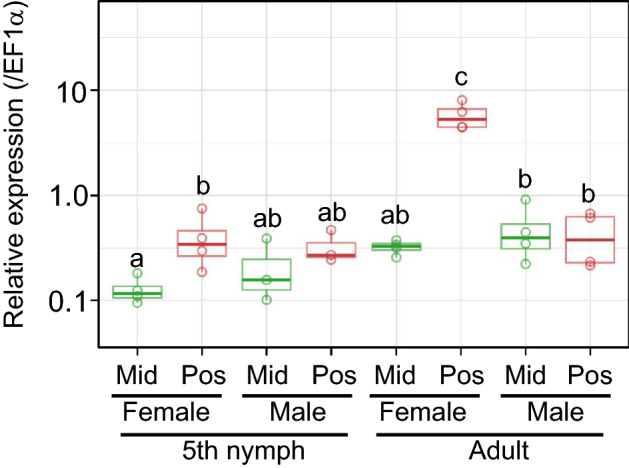
Expression levels of SC mucin gene in the symbiotic midgut. Females and males of fifth instar nymphs and adults were subjected to dissection of the symbiotic midgut, and the middle region (Mid) and the posterior region (Pos) from the samples were individually subjected to RNA extraction and quantitative RT-PCR of the SC mucin gene. Individual quantification values (circles, *N* = 4) are shown with box plots. Different alphabetical letters indicate statistically significant differences (likelihood ratio test for GLM, *P* < 0.05).

**Figure 4:**
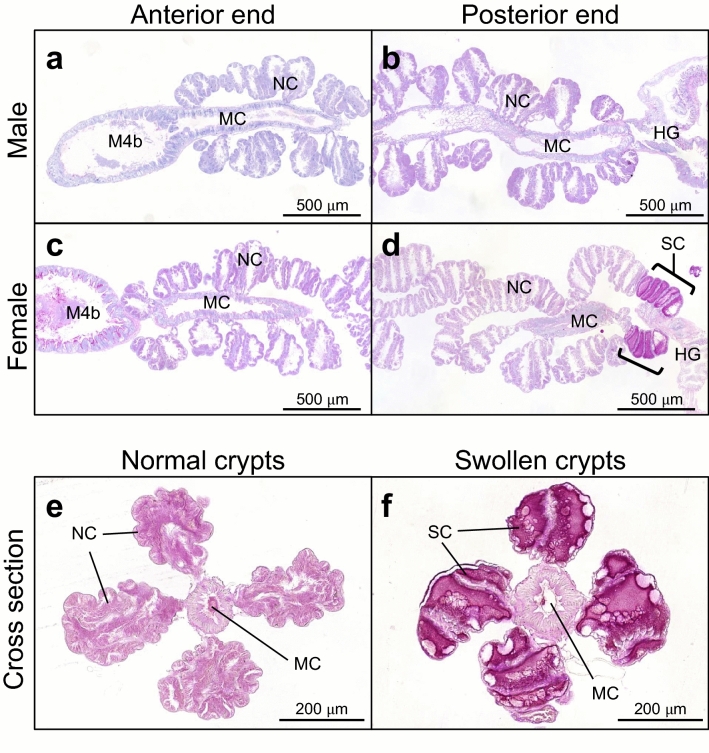
Localization of PAS-positive SC mucin protein in the female-specific swollen crypts of the symbiotic midgut. (**a**,**b**) Longitudinal tissue sections of anterior part (**a**) and posterior part (**b**) of the symbiotic midgut of an adult male. (**c**,**d**) Longitudinal tissue sections of anterior part (**c**) and posterior part (**d**) of the symbiotic midgut of an adult female. (**e**,**f**) Cross tissue sections of the normal crypts (**e**) and the swollen crypts (**f**) of the symbiotic midgut of an adult female. M4b, bulb-like region anterior to M4; NC, normal crypt; SC, swollen crypt; HG, hindgut; MC, midgut main cavity.

**Figure 5:**
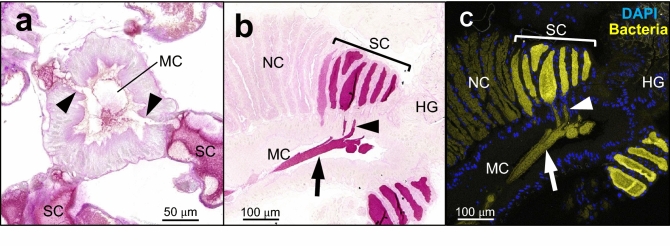
Co-localization of SC mucin protein and symbiotic bacteria in the swollen crypts, and their excretion to the midgut main cavity. (**a**) A cross section of the posterior midgut region of an adult female, on which the narrow ducts connecting the swollen crypts and the midgut main cavity are seen (arrowheads). (**b**) A longitudinal section of the posterior midgut region of an adult female, on which dense PAS signals are seen in the swollen crypts, the narrow ducts, and midgut main cavity, indicating excretion of the SC mucin protein from the swollen crypts through the narrow ducts to the midgut main cavity. (**c**) An adjacent tissue section to (**b**), on which the symbiotic bacteria are visualized by FISH. NC, normal crypts; SC, swollen crypts; MC, midgut main cavity; HG, hindgut.

We examined expression levels of the SC mucin gene in the symbiotic midgut by quantitative RT-PCR using the primers that were designed to amplify both the SC mucin isoforms (Supplementary Fig. S1). In early fifth instar nymphs whose swollen crypts were still undeveloped, the expression levels did not remarkably differ between males and females and between middle and posterior regions of the symbiotic midgut, except that there was a significant difference between the regions in female nymphs (Fig. 3). In mature adults whose swollen crypts were fully developed, the expression levels were significantly higher in the posterior region of female’s symbiotic midgut where the swollen crypts form (Fig. 3). Histochemical inspection of the SC mucin protein by PAS staining visualized strong PAS signals in the cavity of the swollen crypts at the posterior end region of the symbiotic midgut (Fig. 4), verifying the specific expression and localization of the polysaccharide-rich SC mucin protein in the female-specific swollen crypts. The gut epithelium of the swollen crypts was thicker than that of the normal crypts (Figs. 4, 5), presumably reflecting secretion activities there. Detailed histological observations revealed that the swollen crypts were connected to the midgut main cavity via a narrow duct (Fig. 5a). Note that, in adult insects of *P. stali*, the inner cavities of the normal crypts are isolated from the midgut main cavity without connection, plausibly for enabling stable symbiont retention and massive food flow simultaneously^49^. We observed that the SC mucin protein was released from the swollen crypts through the narrow duct into the midgut main cavity (Fig. 5b). FISH on the adjacent tissue sections visualized the localization patterns of the symbiotic bacteria identical to those of the SC mucin protein, being located within the swollen crypts, excreted via the narrow duct, and accumulated in the midgut main cavity (Fig. 5c). These results indicated that the SC mucin protein is specifically produced in female’s swollen crypts, stored in the cavity of the swollen crypts to enclose the symbiotic bacteria, and excreted to the midgut main cavity through the narrow duct together with the symbiotic bacteria.

### Suppression of SC mucin production by RNAi

**Figure 6:**
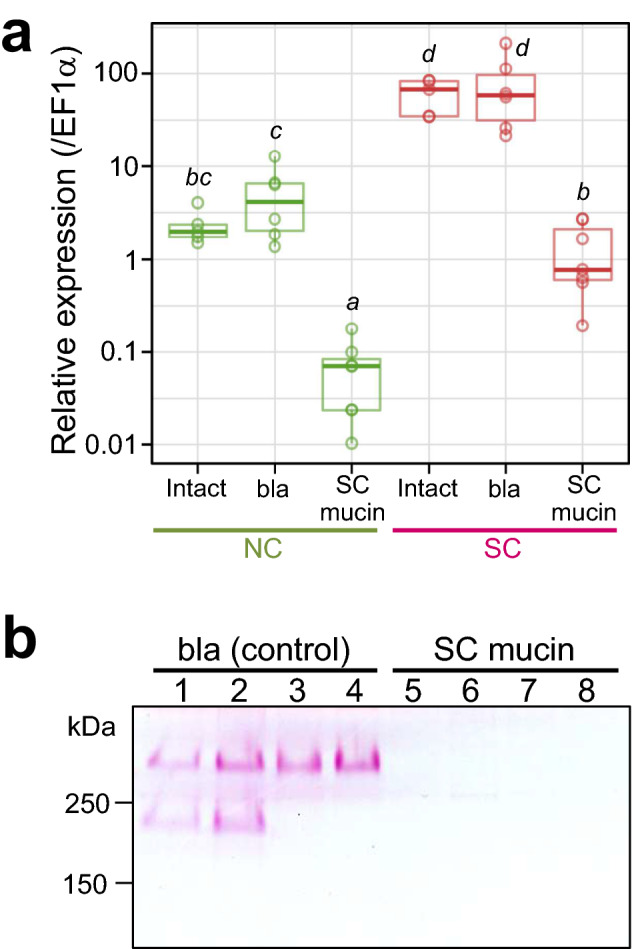
Suppression of SC mucin production by RNAi. (**a**) Suppression of SC mucin gene expression by RNAi. Adult females injected with dsRNA targeting β-lactamase gene (bla), those injected with dsRNA targeting SC mucin gene (SC mucin), and those without injection (Intact) were dissected 14 days after the treatment, and their normal crypts (NC) and swollen crypts (SC) were subjected to quantitative RT-PCR of SC mucin gene expression. Individual quantification values (circles,* N* = 5–7) are shown with box plots. Different alphabetical letters indicate statistically significant differences (likelihood ratio test for GLM, *P* < 0.05). (**b**) Depletion of SC mucin protein in the swollen crypts after RNAi. Adult females 14 days after the treatment were dissected and subjected to SDS-PAGE followed by PAS staining. Lane 1–4 and lanes 5–8 represent different individuals, respectively.

**Figure 7:**
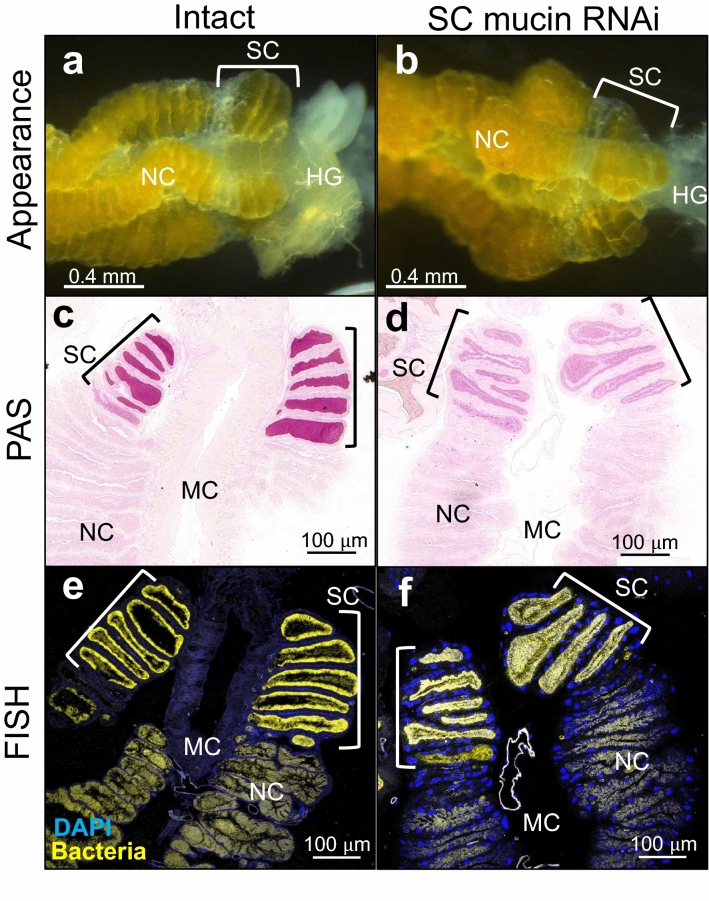
Phenotypic effects of SC mucin RNAi on the symbiotic midgut of adult females. (**a**,**b**) External appearance of the posterior end region of the symbiotic midgut. (**c**,**d**). PAS staining of tissue sections on which localization of SC mucin protein is visualized. (**e**,**f**) FISH of tissue sections on which the symbiotic bacteria are visualized. (**a**,**c**,**e**) Control insects without treatment. (**b**,**d**,**f**). SC mucin RNAi insects. Adult females 14 days after the treatment were dissected and subjected to morphological and histological inspections. NC, normal crypts; SC, swollen crypts; HG, hindgut; MC, midgut main cavity.

Based on these observations, we suspected that the SC mucin may be involved in, and possibly essential for, vertical symbiont transmission via egg surface contamination in *P. stali*. In order to test this hypothesis, we synthesized a dsRNA targeting the 5′ region of the SC mucin gene (Supplementary Fig. S1) and attempted to suppress the SC mucin production by RNAi. Quantitative RT-PCR confirmed that the expression levels of the SC mucin gene were significantly suppressed by injection of the dsRNA into adult females (Fig. 6a). SDS-PAGE and PAS staining showed that the SC mucin protein also drastically decreased in the swollen crypts after the RNAi treatment (Fig. 6b). In the SC mucin RNAi females, enlargement of the terminal crypts was less obvious in comparison with the control females (Fig. 7a,b). Histochemical inspection revealed that PAS stainability of the terminal crypts was clearly reduced in the SC mucin RNAi females (Fig. 7c,d). Meanwhile, FISH observation showed that the symbiont population persisted in the terminal crypts of the SC mucin RNAi females (Fig. 7e,f), indicating that depletion of the SC mucin protein did not conspicuously affect the localization and abundance of the symbiotic bacteria in the terminal crypts.

### Effects of SC mucin depletion on symbiont transmission to offspring

**Figure 8:**
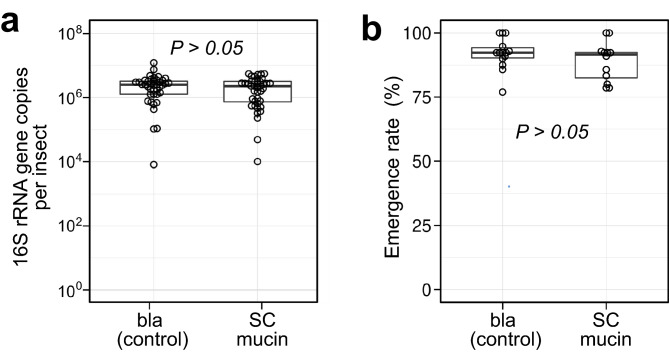
Effects of maternal SC mucin RNAi on vertical symbiont transmission. (**a**) The symbiont titers in the progenies of control (bla dsRNA injected) females and SC mucin dsRNA injected females. For each group, the symbiont titer of 40 s-instar nymphs derived from 10 treated females was assessed by qPCR. (**b**) Adult emergence rate of the offspring. In total, 14 egg masses from 7 control (bla RNAi) females (201 hatched nymphs in total) and 12 egg masses from 6 SC mucin RNAi females (164 hatched nymphs in total) were subjected to nymphal rearing to adulthood to collect the data of adult emergence rate per egg mass. Adults that emerged within a normal growth period (27 days from hatching) were counted. In both (**a**) and (**b**), the difference between the experimental groups was statistically not significant (likelihood ratio test for GLM, *P* > 0.05).

Finally, we evaluated the effects of SC mucin depletion on vertical transmission efficiency of the symbiotic bacteria. Newly emerged adult females were injected with either control (= bla) dsRNA or SC mucin dsRNA. The number of egg masses laid within two weeks per female (5.47 vs 5.87 for control and SC mucin RNAi females respectively, *N* = 15 for each treatment, *P* > 0.05, Tukey T-test), the total number of eggs (89.2 vs 89.7, *P* > 0.05, Tukey T-test), and their hatching rates (80.7% vs 76.1%, *P* > 0.05, a likelihood ratio test for GLM) were not affected by the RNAi treatments. We assessed the symbiont transmission efficiency by quantifying symbiont titers in the young progenies (2nd instar nymphs) derived from the second egg masses. Note that their first egg masses were discarded on account of possible carryover of remnant SC mucin protein. It turned out that the symbiotic bacteria were detected in all the investigated nymphs, and no significant difference was found in the symbiont titers between the two RNAi groups (Fig. 8a). We further assessed the offspring growth rate, because acquisition of the wholesome symbiont is indispensable for normal development in this insect^18^. In both the control group and the SC mucin RNAi group, most of the nymphs grew normally and attained adult emergence rates around 90% within the normal developmental period (Fig. 8b). These results indicate that the maternal SC mucin depletion by RNAi suppressed neither vertical symbiont transmission nor offspring growth.

## Discussion

In this study, we discovered a secretory glycoprotein, SC mucin, as a predominant protein in the female-specific terminal crypts of the symbiotic midgut of the stinkbug *P. stali*. This female-specific organ is relatively small and morphologically not so conspicuous in comparison with the normal crypts (Fig. 1a–c), but our in-depth histochemical observations uncovered not only its morphological and cytological architecture but also its biochemical and molecular specialization (Figs. 4, 5). While previous histological studies have reported the presence of the female-specific swollen crypts in a variety of pentatomid stinkbugs^44–47^, this study is the first to report a molecular aspect of the unique organ found in stinkbug females.

Initially, we found the very dense protein bands, 200 kDa and 400 kDa in estimated size, specific to the swollen crypts on SDS-PAGE gels (Fig. 1d). On the other hand, by molecular cloning and sequencing, we identified 1.1 kbp and 1.4 kbp variant genes encoding the proteins, which correspond to protein sizes of 32.7 kDa and 39.9 kDa, respectively (Fig. 2a, Supplementary Fig. S2a). The stark size discrepancies, 200 kDa vs. 32.7 kDa and 400 kDa vs. 39.9 kDa, are accounted for by massive glycosylation of the proteins, which were verified by strong PAS stainability of the proteins (Fig. 1e), presence of abundant potentially O-glycosylated PTS residues throughout the proteins (Fig. 2a, Supplementary Fig. S2a), and LC–MS analysis of sugars liberated from the hydrolysed proteins (Fig. 2b). We also experienced several difficulties in analyzing the proteins. For example, LC–MS/MS-based protein mapping identified peptides only to the C-terminal region. We attempted to obtain an antibody against the protein by immunizing a rabbit with a synthetic peptide representing a partial protein sequence, but the antibody recognized the synthetic peptide but did not react to the proteins. These difficulties may be attributable to steric hinderance due to bulky polysaccharides that prevent the access of reagents to the polypeptide backbone of the proteins.

Mucins, which are characterized by heavily glycosylated PTS domains, are widespread proteins among animals that form extracellular mucus layers^50,52^. In vertebrates, their protective functions against pathogen infection, desiccation, and physical and chemical injuries have been documented^53^, whereas in insects and other invertebrates, their diversity and functions are still to be fully elucidated on account of their substantial variety in protein structures and expression patterns^54–56^. The SC mucin of *P. stali* possesses a single chitin-binding domain (or peritrophin-A domain) in addition to the PTS domain (Fig. 2a, Supplementary Fig. S2). This protein structure is somewhat reminiscent of the feature of invertebrate intestinal mucins, which are peritrophic membrane-associated glycoproteins called peritrophins^57–60^. The insect peritrophic membrane is an extracellular film consisting of chitins and proteins that surrounds the food boluses in the gut cavity^58,61^. It has been reported that invertebrate intestinal mucins are bound to chitin matrix of the peritrophic membrane via the chitin-binding domains, thereby involved in digestive and protective roles^62–65^. Meanwhile, previous studies have noted that chitinous peritrophic membranes are not found in hemipteran insects including stinkbugs^58,59^. Actually, we observed no chitinous membranes in the symbiotic crypt cavities of *P. stali* (see Figs. 4, 5), suggesting that the SC mucin is unlikely to play a peritrophin-like role, although the possibility that these proteins might share the common deep ancestry, though unrecognizable based on the sequence similarity, cannot be ruled out.

In this study, by making use of histochemistry and FISH, we unequivocally demonstrated co-localization of the SC mucin and the symbiotic bacteria in the swollen crypts (Fig. 4), although this protein is also expressed at quite low but non-negligible levels in the normal crypts (Figs. 3, 6a). We also demonstrated that this glycoprotein is involved in the process of symbiont excretion to the main midgut tract through the special duct structures characteristic to the swollen crypts (Fig. 5). The symbiont-containing mucus released to the posteriormost part of the midgut lumen is expectedly transferred to the egg surface from the anus. Therefore, it is conceivable that the SC mucin may somehow interact with the symbiotic bacteria, and its overexpression in the swollen crypts may be associated with vertical transmission. In this context, it seems relevant that polysaccharide chains of intestinal mucins can be adhesion sites for bacteria that recognize cell-surface sugar chains^61,66^. In *Drosophila*, it was reported that a hemolymphal mucin is used for entrapment of bacteria^67^. In addition to the PTS domain heavily loaded with polysaccharide chains, the SC mucin contains a chitin-binding domain despite the apparent absence of chitinous structures in the midgut symbiotic organ. Notably, chitin-binding domains of some antimicrobial peptides are needed for interaction with bacteria via binding to their surface polysaccharides^68–70^. Hence, although speculative, the polysaccharide coat and/or the chitin-binding domain of the SC mucin might serve as possible recognition and attachment sites for the symbiotic bacteria. In general, mucin glycoproteins possess high water holding capacity^53^ and high resistance against digestive enzymes^59^. These features of mucin proteins may be beneficial for the symbiotic bacteria that are smeared on the egg surface for vertical transmission where they suffer desiccation, UV irradiation and other environmental stresses. Another possible function of the SC mucin may be as an organic carbon and nitrogen source for proliferation of the symbiotic bacteria. Upon every oviposition, the symbiotic bacteria in the swollen crypts must be excreted and consumed for transfer to the egg surface. Highly glycosylated mucin proteins can be an ideal energy source for symbiotic and commensal gut bacteria^53,71^. The enhanced supply of the SC mucin may support replenishment of the symbiont population in the swollen crypts.

By injecting SC mucin dsRNA, we successfully generated adult females whose swollen crypts were depleted of the SC mucin by RNAi (Figs. 6, 7). Considering the specific localization, abundance, and co-localization with the symbiotic bacteria, we expected that the SC mucin depletion would negatively influence vertical transmission of the symbiotic bacteria. Contrary to the expectation, however, newborn nymphs from the egg masses laid by the SC mucin RNAi females acquired a substantial amount of the symbiotic bacteria and attained high adult emergence rates that are comparable to those of control newborn nymphs (Fig. 8). There are several possible reasons as to why the SC mucin depletion did not suppress the vertical symbiont transmission. Firstly, considering that vertical transmission is a pivotal process for sustaining the obligate symbiosis, multiple factors may be involved in vertical transmission of the symbiotic bacteria, and the role of the SC mucin may be complemented by other factors. We are now surveying such factors by RNA sequencing of the female-specific swollen crypts in combination with RNAi knockdown of candidate genes. Secondly, the RNAi treatment may have certainly suppressed the SC mucin production but not completely, and the remnant protein may be sufficient for ensuring vertical symbiont transmission. Thirdly, the SC mucin may certainly play some roles, but the roles are not essential for successful vertical transmission of the symbiotic bacteria. It should be noted that our experiments are conducted under an unnatural laboratory condition. In the field, eggs of *P. stali* are normally laid on host plant leaves, and symbiont-containing secretion on the eggs must experience a variety of environmental challenges, including solar radiation, washout by rain, predation, desiccation, and invasion of microbial contaminants. Considering the potential protective functions of mucin-type glycoproteins as discussed above, we suspect that the SC mucin may function by embedding the symbiotic bacteria and protecting them against environmental stresses. Alternatively, the substantial amount of SC mucin produced in the swollen crypts may contribute to constituting voluminous mucus to facilitate the excretion and smearing of the symbiotic bacteria, but it may be not essential for symbiont transmission and survival. So far, we could not detect SC mucin on the egg surface, primarily due to the difficulty to construct a specific antibody as mentioned above. To explore the full transmission route of symbiont-containing secretion from the swollen crypts to the progenies and to identify the functional component for vertical transmission are our future subjects.

In intimate host-symbiont associations, both the host and the symbiont are integrated into an almost inseparable biological entity, where the symbiont cannot survive without the host and vice versa. Such symbiotic bacteria tend to exhibit genome reduction and gene losses, which lead to their incapability of independent survival and proliferation^9,10^. This particularly matters for symbiont-dependent stinkbugs, because their extracellular gut symbiotic bacteria have to be excreted and spend some time from oviposition to egg hatching outside the host for establishing vertical transmission to offspring^5,35^. Recent studies have unveiled that such stinkbugs have evolved special molecules that are produced in female-specific organs, excreted upon or after oviposition, and ensuring survival of fragile symbiotic bacteria outside the host. In stinkbugs of the family Plataspidae, the genome reduced symbiont *Ishikawaella* is embedded in a novel secretion protein with an odorant binding protein motif, encased in “capsules” covered with chitinous shell, and deposited beside the eggs, where the protein is essential for vertical symbiont transmission^34^. In stinkbugs of the family Urostylididae, the genome reduced symbiont *Tachikawaea* is implemented in voluminous galactose polymer gel, and the symbiont-containing “jelly” covers the eggs, protects them against desiccation and other environmental stresses, and serves as food source and symbiont inoculum for the nymphs^21^. In *P. stali* belonging to the family Pentatomidae, we identified a novel mucin protein that is preferentially produced by the female-specific swollen crypts and associated with the genome-eroded uncultivable symbiont *Pantoea*, although its biological role is still to be elucidated. In this way, different stinkbug groups have established intimate mutualistic associations with different bacteria, and have co-opted different molecular factors for sustaining the symbiotic associations, which highlight the dynamic aspect of host-symbiont co-evolution that can be embodied through a variety of evolutionary trajectories.

## Methods

### Insect materials and preparation of symbiotic midgut

We used a laboratory-maintained strain of *P. stali*, which was originally collected in Tsukuba, Ibaraki, Japan. This strain is associated with an obligatory uncultivable gut symbiont, *Pantoea* sp. A^18^, with no coexisting facultative symbionts. They were reared on raw peanuts and water supplemented with 0.05% ascorbic acid at 25 °C under a 16 h light and 8 h dark photoperiodic cycle. To obtain the symbiotic midgut used for the experiments, ice-anesthetized adult insects were dissected in a phosphate buffered saline (PBS: 137 mM NaCl, 8.10 mM Na_2_HPO_4_, 2.68 mM KCl, 1.47 mM KH_2_PO_4_, pH 7.4) using fine forceps. The dissected midgut was washed several times with sterilized PBS and divided into the normal crypt region and the swollen crypt region using an ophthalmic razor blade.

### SDS-PAGE

The dissected normal crypts and the swollen crypts were subjected to a sodium dodecyl sulfate–polyacrylamide gel electrophoresis (SDS-PAGE) analysis. Each tissue was homogenized in 50 μL of an SDS-PAGE sample buffer (57.7 mM Tris–HCl, 2% SDS, 10% (v/v) glycerol, 5% (v/v) 2-mercaptoethanol, 0.002% bromophenol blue, pH 6.8), and heated at 100 °C for 3 min. For electrophoresis, we used 5% or 10% polyacrylamide gels. The gel was stained with CBB-solution (250 mg/L Coomassie Brilliant Blue R-250, 5% (v/v) methanol, 7.5% (v/v) acetic acid). To detect glycoproteins, some SDS-PAGE gels were subjected to periodic acid-Shiff (PAS) staining, in which the gels were treated with 1% periodic acid and 3% acetic acid solution for 50 min, and then incubated in cold-Schiff reagent (Fujifilm) for 30 min to visualize polysaccharides in reddish violet.

### LC–MS for protein identification

After CBB staining of SDS-PAGE gels, some protein bands were cut and stored at − 80 °C for protein identification using liquid chromatography and mass spectrometry (LC–MS)^72^. The gel pieces were subjected to disulfide reduction with 10 mM dithiothreitol in 25 mM ammonium bicarbonate (ABC) solution, and then to alkylation with 55 mM 2-iodoacetamide in 25 mM ABC solution. After drying, the gel pieces were infiltrated with a digesting solution containing 10 μg/mL sequence grade modified trypsin (Promega), and incubated at 36 °C for 16 h. The digested peptides were extracted with 5% (v/v) formic acid- and 50% (v/v) acetonitrile-containing water, and desalted using a solid-phase extraction column (GL Tip-SDB, GL Science). Mass analyses were performed using a liquid chromatography system (Prominence, Shimadzu) coupled with an ion-trap mass spectrometer (LCQ-Fleet, Thermo) equipping an electrospray ionization source in a positive ion detection mode. Peptides were separated using an FC-ODS column (2 mm i.d. × 150 mm, Shimadzu) in a gradient elution of acetonitrile against 0.1% formic acid-containing water at a flow rate of 0.2 mL/min. The obtained precursor/fragment mass data were subjected to protein identification using an MS/MS ion search algorism on Mascot Server (v2.7, Matrix Science). For reference, we built a protein sequence database of *P. stali* based on RNA sequencing data prepared from the whole midgut symbiotic region. The total RNA was extracted using RNAiso Plus (Takara) and purified with RNeasy columns (QIAGEN). The libraries were constructed using TruSeq stranded mRNA Kit (Illumina) and sequenced using Hiseq 3000 (Illumina). The sequence data were subjected to *de-novo* assembling using Trinity v2.11^73^, and converted to protein sequences by TransDecoder v5.5.0^74^. The RNA sequencing data were deposited in the DNA Data Bank of Japan under accession numbers DRX303863-5.

### cDNA cloning and sequencing

To determine full-length sequences of the genes encoding the swollen crypt-specific proteins, total RNA was extracted from the symbiotic midgut as described above. Full-length cDNA libraries were built using SMART cDNA Library Construction Kit (Clontech), and the 5′- and 3′-terminal fragments of the target genes were amplified using the primers (5RACE_R, 3RACE_F) shown in Supplementary Fig. S1. The sequences of these cDNA fragments were determined using BigDye Terminator v3.1 Cycle Sequencing Kit (Life Technologies) and a DNA sequencer (ABI3130x, Applied Biosystems). To resolve the variants of the swollen crypt-specific proteins, we verified the length polymorphism of the gene by amplifying mid-sections using the primers (SC_F1, SC_F2, SC_R1, SC_R2) indicated in Supplementary Fig. S1, and sequenced them as described above. The sequencing data were deposited in the DNA Data Bank of Japan under accession numbers LC661891-2.

### Sugar component analysis

To determine sugar compositions associated with the target proteins, the protein bands were excised from SDS-PAGE gels after zinc-imidazole negative staining^75^. The gel pieces were ground in an extraction buffer (1% SDS in 20 mM Tris–HCl, pH 8.0) and incubated for 16 h to allow protein diffusion. The extracted proteins were retrieved using an ultrafiltration column (Amicon Ultra, 10 kDa cut off, Merck), precipitated by adding acetone, and lyophilized. The samples were hydrolysed by incubating at 110 °C for 4 h in 2 M trifluoroacetic acid. After drying, the hydrolysate was subjected to N-acetylation using acetic anhydride and derivatized with 1-phenyl-3-methyl-5-pyrazolone^76^. Identification and quantification of the derivatized monosaccharides were performed using the above-mentioned LC/MS system. InertSustain C18 column (2 mm i.d. × 150 mm, GL Science) was used at a flow rate of 0.2 mL/min under a gradient elution of 10 mM ammonium formate solution and acetonitrile.

### Quantification of gene expression levels

To quantify gene expression levels, we prepared mRNA samples from the dissected symbiotic midgut. The total RNAs were extracted and purified using RNAiso Plus (Takara) and RNeasy kit (QIAGEN), and reverse-transcribed using ReverTra Ace (TOYOBO). Expression levels of target genes were quantified by real-time quantitative PCR using KAPA SYBR Fast qPCR kit (Nippon Genetics) and MX3000P (Stratagene). We calculated the gene expression levels based on Ct values of standard plasmids (pT7Blue T-vector, Merck) carrying the target fragment sequences and normalized them to the constitutive gene Elongation Factor 1 alpha (EF1α). The gene-specific primer pairs (SC_qF, SC_qR, PsEF1a_qF, PsEF1a_qR) used for the quantitative PCR are shown in Supplementary Fig. S1. The gene expression levels were quantified and compared between a variety of combinations of developmental stages, sexes, and M4 regions (Fig. 3) or combinations of RNAi treatments and M4 regions (Fig. 6a). To ensure flat and simple comparisons of the gene expression levels among these treatment groups, we adopted single one-factor GLM rather than multi-factor GLM for statistics.

### Histological observation

The dissected symbiotic midgut samples were fixed with 4% paraformaldehyde in PBS. After washing with PBST (0.1% Triton-X containing PBS), the samples were dehydrated with a graded alcohol series and embedded in Technovit 7100 or 8100 resin (Kulzer). The embedded samples were processed into 2 μm sections using a microtome (RM2255, Leica). The sections were fixed on glass slides and subjected to PAS staining or fluorescent in situ hybridization (FISH). For PAS staining, the sections were treated with 0.5% periodic acid for 10 min and then cold-Schiff reagent (Fujifilm) for 2 min. After counterstaining with hematoxylin (Fujifilm), the sections were sealed in a mounting agent (Entellan New, Merck) and observed under a light microscope. FISH was performed essentially as described previously^49^ using an oligonucleotide probe EUB917 conjugated with Alexa Fluor 647 fluorescent dye that was designed for eubacterium detection^77^. Counter fluorescent staining was conducted with 4′,6-diamidino-2-phenylindole (DAPI) for nucleic acid visualization.

### RNA interference

RNA interference (RNAi) was performed as described previously^78^. A 331 bp fragment located in the shared region of the two isoforms was amplified using the primers (SCdsR_F, SCdsR_R) shown in Supplementary Fig. S1 and cloned into pT7Blue T-vector (Merck). The double-stranded RNA (dsRNA) was synthesized using MEGAscript RNAi Kit (Thermo Fisher Scientific). Females one day after adult emergence were injected with 1 μL of the 200 ng/μL dsRNA solution using a glass capillary from the intersegmental membrane between head and prothorax. As a control treatment, dsRNA of β-lactamase (bla) gene fragment was injected in the same manner. To assess the transmission rates of symbiotic bacteria, the treated adult females were allowed to lay egg masses for two weeks. Note that most females started to lay eggs after about a week. For direct detection of the transmitted symbiont, we performed qPCR using specific primers for the 16S rRNA gene of symbiont A^18^. The offspring DNA samples were extracted from the whole body of second instar nymphs at the day of ecdysis using DNA MINI Kit (QIAGEN). qPCR was performed as mentioned above, but the different reagent [KOD One (TOYOBO) supplemented with SYBR green I (BioWhittaker Molecular Applications) fluorescent dye^79^] was used. It should be noted that, when uninfected nymphs emerging from formaldehyde-sterilized eggs^18,80^ were subjected to qPCR, no detectable amplification was observed within 32 PCR cycles in this procedure (0/8 nymphs). We also assessed the offspring growth rate as another index of infection status. The progenies were aseptically reared in plastic dishes with sterilized water and raw peanuts^80^, and recorded the adult emergence rate within a normal growth period (27 days from hatching). We used second egg masses for the qPCR and second and third egg masses for the rearing experiment. Firstly-laid egg masses were not used in order to exclude the possibility of remnant effects of target proteins.

## Supplementary Information

Supplementary Figures.
