# Supplementary material for: A mucin protein predominantly expressed in the female-specific symbiotic organ of the stinkbug Plautia stali
Source: Sci Rep. 2022 May 11;12:7782. doi: 10.1038/s41598-022-11895-1 (PMC9095716; doi:10.1038/s41598-022-11895-1)
Supplement: Supplementary file 1 — Supplementary Figures. [file 41598_2022_11895_MOESM1_ESM.pdf]

a

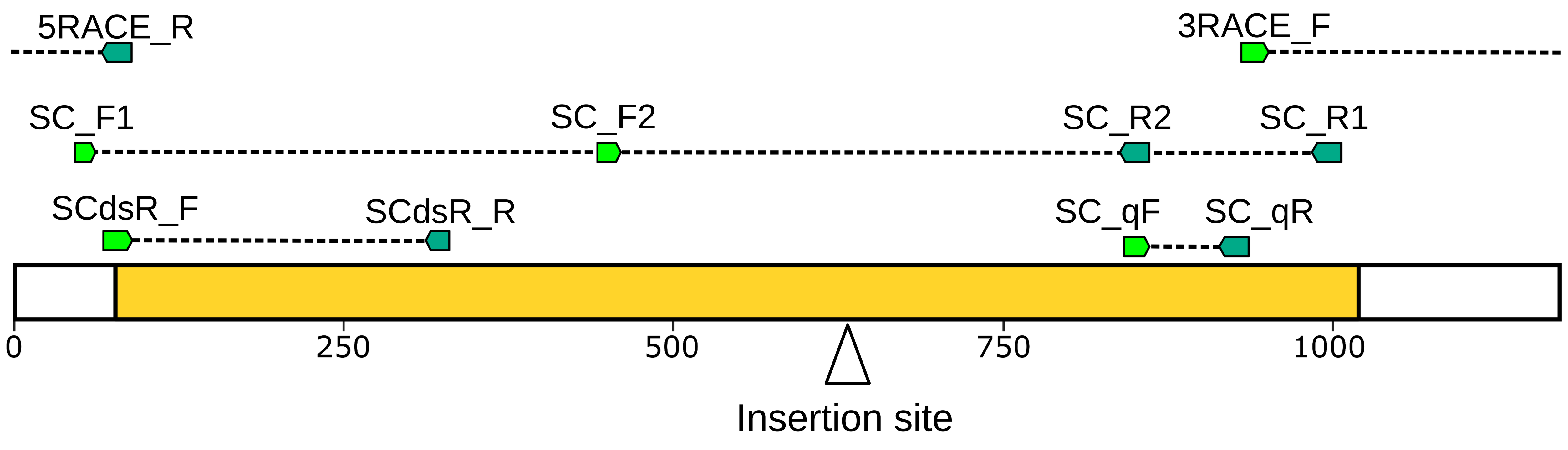

b

Name	Sequence (5' -> 3')	Application
5RACE_R	GATGTAGCATCATCTTTCCTTCGAG	Cloning/sequencing
3RACE_F	GGCTTCTGGGTCCACAGGTCATG	Cloning/sequencing
SC_F1	CATTGGAACCTGCGCCAC	Cloning/sequencing
SC_F2	TCGGACCAGGATCCTCAACT	Cloning/sequencing
SC_R2	CTCGGGTAATCAGTCGTTGTGGGAG	Cloning/sequencing
SC_R1	GGCTTCTGGGTCCACAGGTCATG	Cloning/sequencing
SCdsR_F	CGAAGGAAAGATGATGCTACATC	dsRNA construction
SCdsR_R	GTACCCGTCGTGTTGTCATG	dsRNA construction
SC_qF	CAAGTACCCCAAGAGCTCGTTCG	qPCR
SC_qR	CAGAAGCCATCTTTGTCACATTCCA	qPCR
PsEF1a_qF	AGAATTGCGCCGAGGTTATGTTG	qPCR
PsEF1a_qR	TGACCTGGGCAGTGAAGTCAG	qPCR

Supplementary Figure S1. Primers used in this study. (**a**) Schematic representation of primer positions on the *SC-mucin* gene. This figure indicates the shorter isoform, while the longer one has a 267 bp insertion sequence at the site indicated by the white triangle. (**b**) The sequence list of the primers.

Figure S1

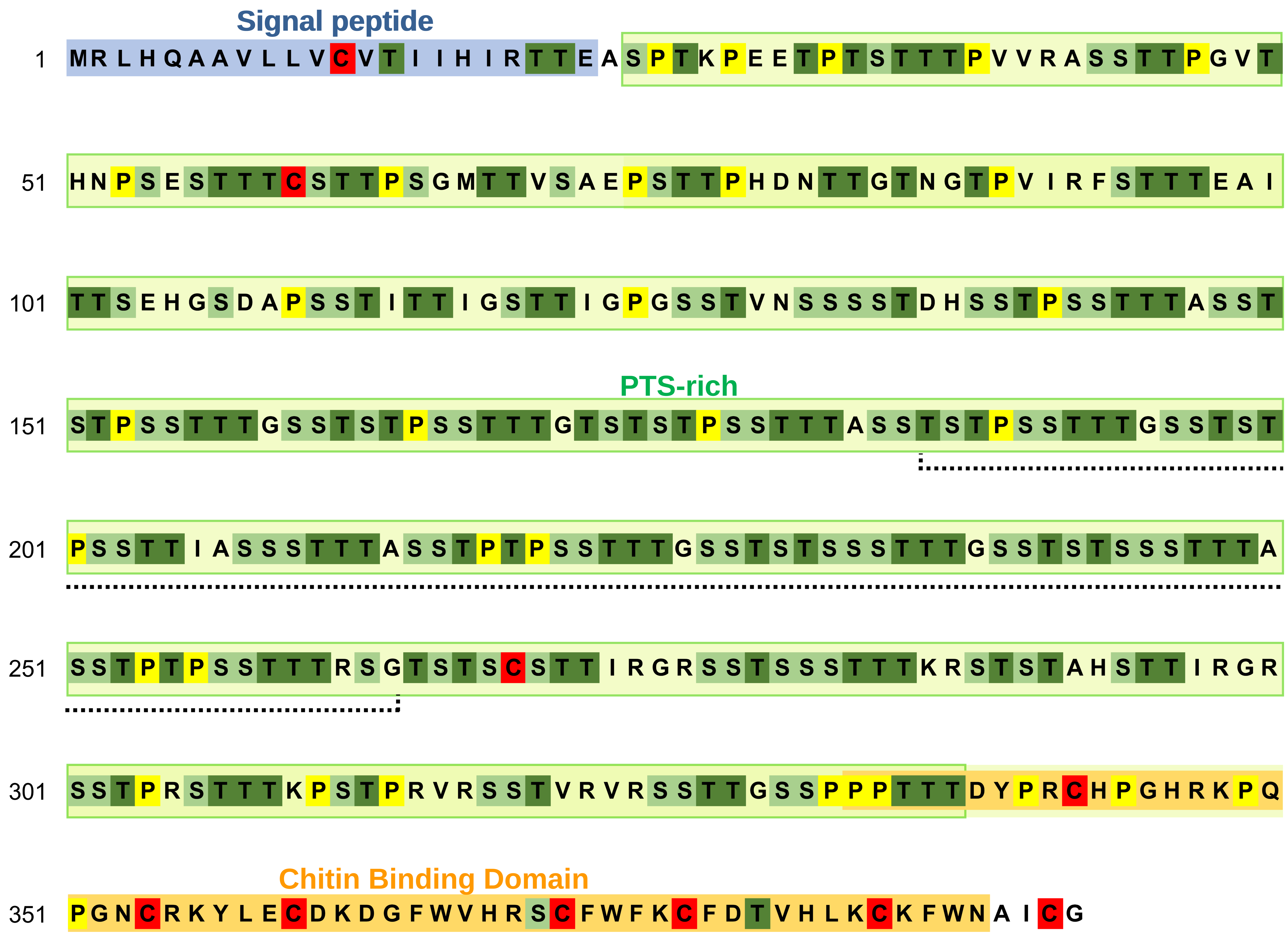

Supplementary Figure S2. Amino acid sequence of longer isoform of SC mucin protein. Signal peptide, Pro-Thr-Ser (PTS)-rich domain, and chitin-binding domain are shown in blue, green, and orange boxes, respectively. Potential O-glycosylated amino acid residues are highlighted in yellow for proline, green for threonine, and light green for serine, while cysteine is marked in red. The broken line indicates the insertion sequence specific to the longer isoform.

.

Figure S2

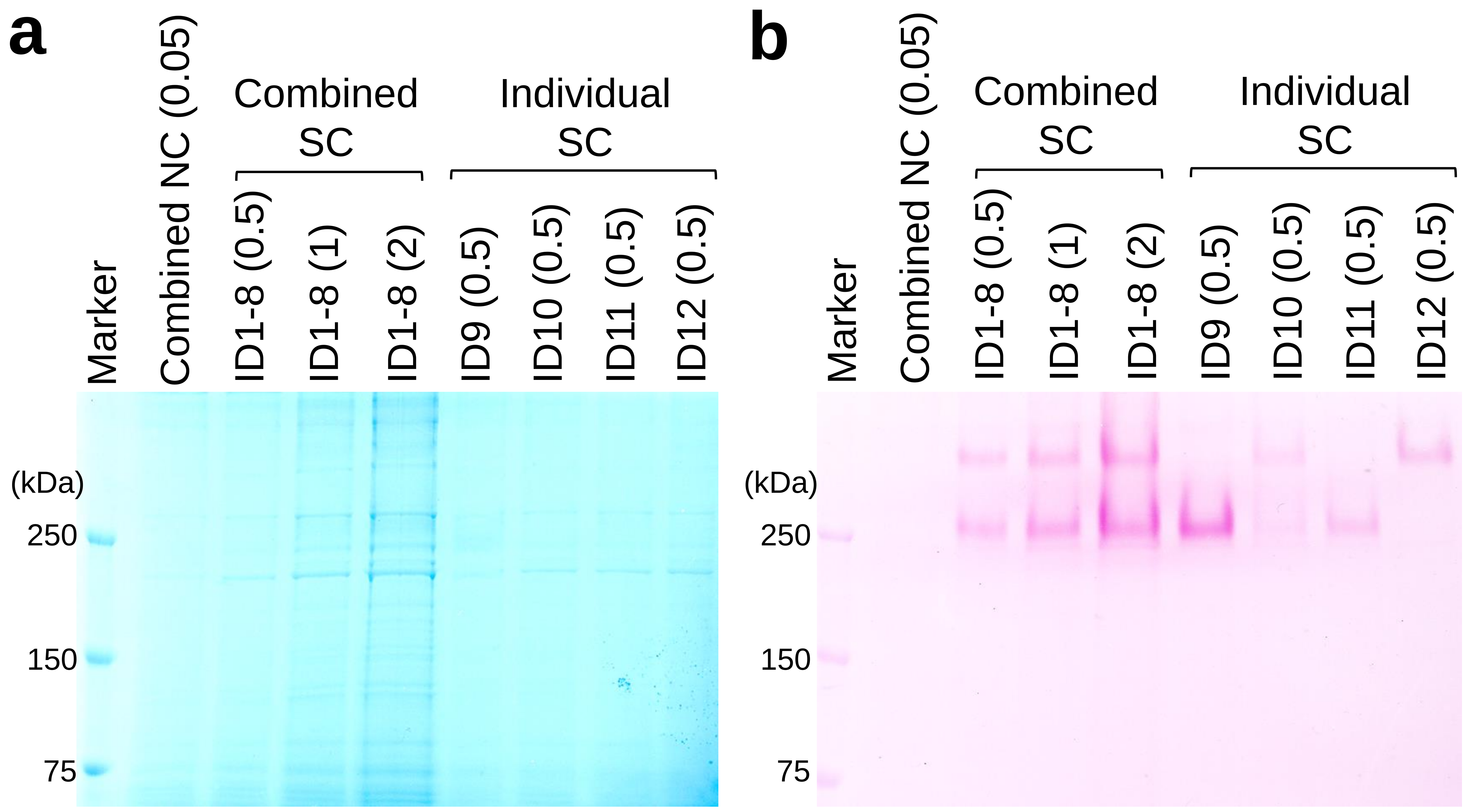

Supplementary Figure S3. Size polymorphism of SC mucin protein at the individual level. (**a**) CBB-stained SDS-PAGE gel. (**b**) PAS-stained SDS-PAGE gel. From eight adult females, the normal crypt (NC) region and the swollen crypt (SC) region were dissected and subjected to SDS-PAGE either individually or in combination. Abbreviations: "Individual", the sample is derived from a single insect; "Combined", the sample represents an equal mixture of the samples from eight insects; "ID1", individual no. 1; "ID1-8", mixture of individuals nos. 1-8; "(0.5)", sample amount equivalent to 1/2 insect. For example, "Individual SC, ID1(0.5)" indicates a SC protein sample from individual no. 1 equivalent to 1/2 insect in quantity, whereas "Combined SC, ID1-8(2)" indicates a mixed SC protein sample from individuals nos. 1-8 equivalent to 2 insects in quantity.

Figure S3
